# Supplementary material for: Association between oral microbiome and seven types of cancers in East Asian population: a two-sample Mendelian randomization analysis
Source: Front Mol Biosci. 2023 Nov 21;10:1327893. doi: 10.3389/fmolb.2023.1327893 (PMC10702768; doi:10.3389/fmolb.2023.1327893)
Supplement: Supplementary file 1 [file DataSheet1.ZIP › Supplementary Materials/MR plots for tongue/tongue═╝/Pancreatic cancer/╥╚╧┘░⌐_╔╕╤í╜ß╣√/pheno.2029_to_pancreatic cancer_leave_one_out.pdf]

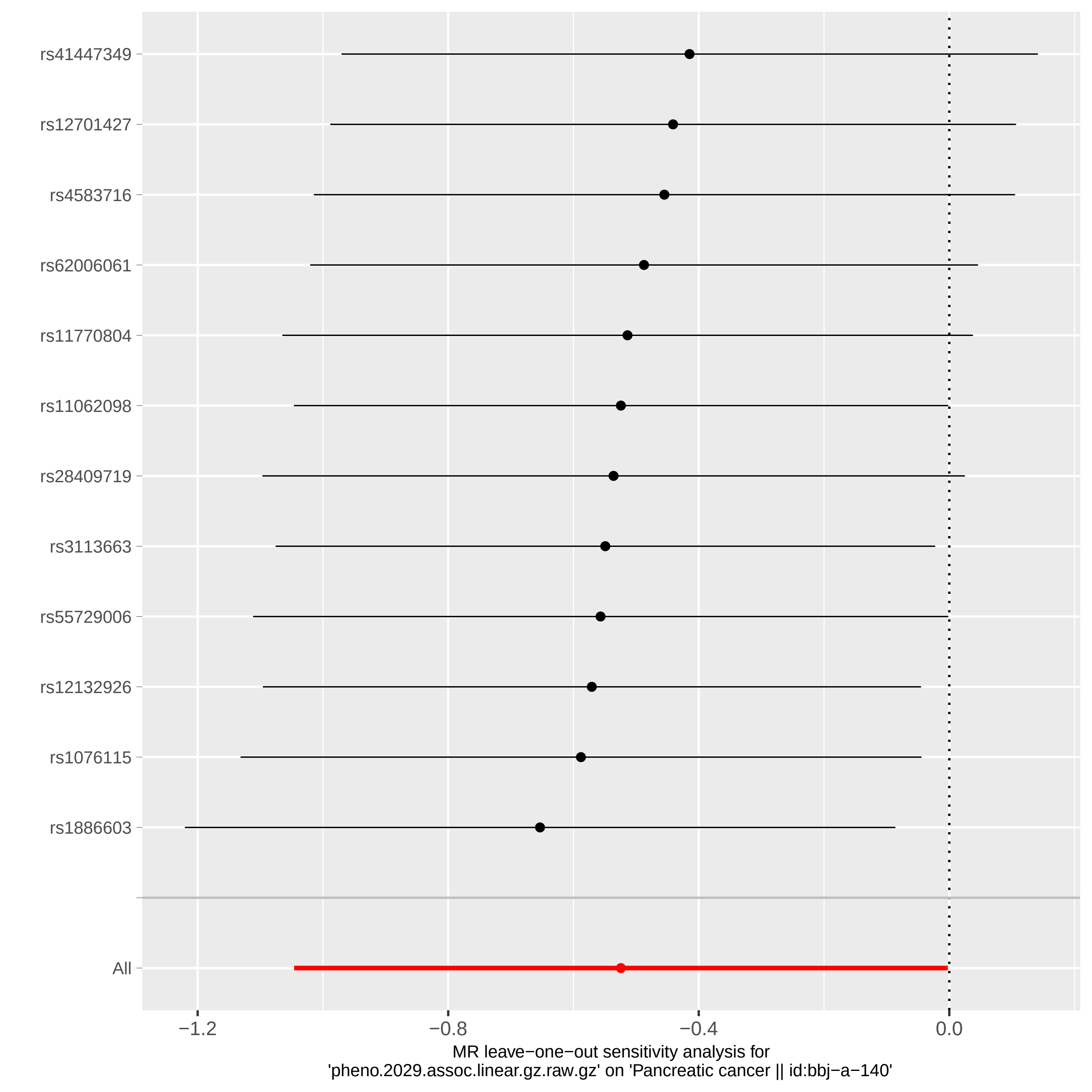
rs41447349
rs12701427
rs4583716
rs62006061
rs11770804
rs11062098
rs28409719
rs3113663
rs55729006
rs12132926
rs1076115
rs1886603
All
−1.2
−0.8
−0.4
0.0
MR leave−one−out sensitivity analysis for
'pheno.2029.assoc.linear.gz.raw.gz' on 'Pancreatic cancer || id:bbj−a−140'
